# Bis(1*H*-pyrazole-κ*N* ^2^)bis­(2,4,6-tri­isopropyl­benzoato-κ*O*)cobalt(II)

**DOI:** 10.1107/S1600536810039553

**Published:** 2010-10-13

**Authors:** Cristian G. Hrib, Steffen Blaurock, Frank T. Edelmann

**Affiliations:** a Chemisches Institut der Otto-von-Guericke-Universität, Universitätsplatz 2, D-39116 Magdeburg, Germany

## Abstract

The title compound, [Co(C_16_H_23_O_2_)_2_(C_3_H_4_N_2_)_2_] or (C_3_H_4_N_2_)_2_Co(O_2_CC_6_H_2_^*i*^Pr_3_-2,4,6), is a rare example of a tetra­coordinate cobalt(II) carboxyl­ate stabilized by ancillary *N*-heterocyclic ligands. The Co(II) ion resides on a crystallographic twofold axis so that the asymmetric unit comprises one half-mol­ecule. Due to the steric bulk of the 2,4,6-triisopropyl­phenyl substituents, the carboxyl­ate ligands are both coordinated in a monodentate fashion despite the low coordination number. The coordination geometry around the central Co(II) ion is distorted tetra­hedral with angles at Co ranging from 92.27 (18)° to 121.08 (14)°.

## Related literature

For cobalt(II) carboxyl­ate complexes containing *N*-coordin­ated heterocyclic ligands, see: Manhas *et al.* (1975 ▶); Catterick & Thornton (1976 ▶); Kumar & Gandotra (1980*a* ▶,*b* ▶); Kumar & Bajju (1999 ▶); Ju *et al.* (2006 ▶); Karmakar *et al.* (2007 ▶). Normally the carboxyl­ate anions are either bidentate or bridging. For an exception in which the benzoate ligands are coordinated in a monodentate fashion, see: Hökelek & Necefouğlu (1999 ▶). Inter­esting supra­molecular structures have also been reported, see: Boldog *et al.* (2001 ▶).
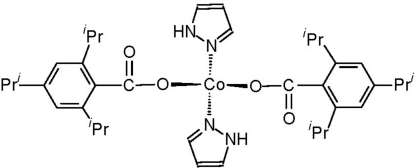


## Experimental

### 

#### Crystal data

[Co(C_16_H_23_O_2_)_2_(C_3_H_4_N_2_)_2_]
*M* *_r_* = 689.78
Orthorhombic, 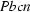

*a* = 9.6146 (19) Å
*b* = 12.792 (3) Å
*c* = 31.275 (6) Å
*V* = 3846.5 (13) Å^3^
*Z* = 4
Mo *K*α radiation
μ = 0.49 mm^−1^
*T* = 153 K
0.80 × 0.50 × 0.10 mm

#### Data collection

Stoe STADI4 diffractometer
Absorption correction: ψ scan (North *et al.*, 1968 ▶) *T*_min_ = 0.696, *T*_max_ = 0.953
6256 measured reflections
3378 independent reflections
2073 reflections with *I* > 2σ(*I*)
*R*_int_ = 0.071
3 standard reflections every 120 min  intensity decay: 3%

#### Refinement

*R*[*F* ^2^ > 2σ(*F* ^2^)] = 0.067
*wR*(*F* ^2^) = 0.173
*S* = 1.09
3378 reflections
213 parameters
H-atom parameters constrained
Δρ_max_ = 0.43 e Å^−3^
Δρ_min_ = −0.48 e Å^−3^

### 

Data collection: *DIF4* (Stoe & Cie, 1992 ▶); cell refinement: *DIF4*; data reduction: *REDU4* (Stoe & Cie, 1992 ▶); program(s) used to solve structure: *SHELXS97* (Sheldrick, 2008 ▶); program(s) used to refine structure: *SHELXL97* (Sheldrick, 2008 ▶); molecular graphics: *XP* (Sheldrick, 2008 ▶); software used to prepare material for publication: *SHELXL97*.

## Supplementary Material

Crystal structure: contains datablocks I, New_Global_Publ_Block. DOI: 10.1107/S1600536810039553/fj2344sup1.cif

Structure factors: contains datablocks I. DOI: 10.1107/S1600536810039553/fj2344Isup2.hkl

Additional supplementary materials:  crystallographic information; 3D view; checkCIF report

## Figures and Tables

**Fig. 1.**
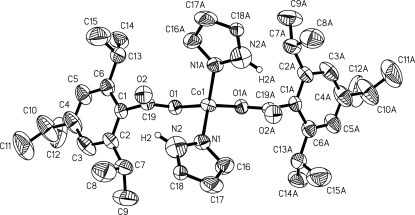
The molecule of the title compound in the crystal. Thermal ellipsoids represent 50% probability levels.

## supplementary crystallographic information

### Comment

Cobalt(II) carboxylate complexes containing *N*-coordinated heterocyclic ligands have been the subject of detailed structural investigations in the past [Manhas *et al.* (1975); Catterick *et al.* (1976); Kumar *et al.* (1980a,b, 1999); Ju *et al.* (2006); Karmakar *et al.* (2007)]. The most frequently employed co-ligands are pyridine derivatives. Normally the carboxylate anions are either bidentate or bridging. A notable exception is the octahedral complex *trans*-diaqua-bis(benzoato-*O*)-bis(nicotinamide-N1)cobalt(II), in which the benzoate ligands are coordinated in a monodentate fashion [Hökelek *et al.* (1999)]. Interesting supramolecular structures have have also been reported in this chemistry [Boldog *et al.* (2001)]. These compounds contained the heterocyclic co-ligand 3,3',5,5'-tetramethyl-4,4'-bipyrazolyl. The title compound, which contains unsubstituted pyrazole as co-ligand, was obtained in small amounts from a reaction of cobalt(II) hydroxide with 2,4,6-triisopropylbenzoic acid in aqueous solution in the presence of pyrazole. The coordination geometry around the central cobalt atom is distorted tetrahedral. Due to the steric bulk of the 2,4,6-triisopropylphenyl substituents the carboxylate ligands in the title compound are monodentate despite the low coordination number of 4 around Co.

### Experimental

Small amounts of blue single crystals of the title compound were obtained from a reaction of cobalt(II) hydroxide with 2,4,6-triisopropylbenzoic acid in aqueous solution in the presence of pyrazole.

### Refinement

The hydrogen atoms were included using a riding model, with N2—H2 = 0.88 Å, aromatic C—H = 0.95 Å, methyn C—H = 1.00 Å [*U*_iso_(H) = 1.2Ueq(C)] and methyl C—H = 0.98 Å [*U*_iso_(H) = 1.5Ueq(C)].

### Crystal data

**Table:**

[Co(C_16_H_23_O_2_)_2_(C_3_H_4_N_2_)_2_]	*F*(000) = 1476
*M**_r_* = 689.78	*D*_x_ = 1.191 Mg m^−^^3^
Orthorhombic, *P**b**c**n*	Mo *K*α radiation, λ = 0.71073 Å
Hall symbol: -P 2n 2ab	Cell parameters from 25 reflections
*a* = 9.6146 (19) Å	θ = 15–25°
*b* = 12.792 (3) Å	µ = 0.49 mm^−^^1^
*c* = 31.275 (6) Å	*T* = 153 K
*V* = 3846.5 (13) Å^3^	Platelet, violet
*Z* = 4	0.80 × 0.50 × 0.10 mm

### Data collection

**Table:**

Stoe STADI4 diffractometer	2073 reflections with *I* > 2σ(*I*)
Radiation source: fine-focus sealed tube	*R*_int_ = 0.071
graphite	θ_max_ = 25.0°, θ_min_ = 2.5°
ω–θ–scans	*h* = −11→11
Absorption correction: ψ scan (North *et al.*, 1968)	*k* = −15→0
*T*_min_ = 0.696, *T*_max_ = 0.953	*l* = −37→0
6256 measured reflections	3 standard reflections every 120 min
3378 independent reflections	intensity decay: 3%

### Refinement

**Table:**

Refinement on *F*^2^	Primary atom site location: structure-invariant direct methods
Least-squares matrix: full	Secondary atom site location: difference Fourier map
*R*[*F*^2^ > 2σ(*F*^2^)] = 0.067	Hydrogen site location: inferred from neighbouring sites
*wR*(*F*^2^) = 0.173	H-atom parameters constrained
*S* = 1.09	*w* = 1/[σ^2^(*F*_o_^2^) + (0.0566*P*)^2^ + 5.530*P*] where *P* = (*F*_o_^2^ + 2*F*_c_^2^)/3
3378 reflections	(Δ/σ)_max_ < 0.001
213 parameters	Δρ_max_ = 0.43 e Å^−^^3^
0 restraints	Δρ_min_ = −0.48 e Å^−^^3^

### Special details

**Table:**

Geometry. All e.s.d.'s (except the e.s.d. in the dihedral angle between two l.s. planes) are estimated using the full covariance matrix. The cell e.s.d.'s are taken into account individually in the estimation of e.s.d.'s in distances, angles and torsion angles; correlations between e.s.d.'s in cell parameters are only used when they are defined by crystal symmetry. An approximate (isotropic) treatment of cell e.s.d.'s is used for estimating e.s.d.'s involving l.s. planes.
Refinement. Refinement of *F*^2^ against ALL reflections. The weighted *R*-factor *wR* and goodness of fit *S* are based on *F*^2^, conventional *R*-factors *R* are based on *F*, with *F* set to zero for negative *F*^2^. The threshold expression of *F*^2^ > σ(*F*^2^) is used only for calculating *R*-factors(gt) *etc*. and is not relevant to the choice of reflections for refinement. *R*-factors based on *F*^2^ are statistically about twice as large as those based on *F*, and *R*- factors based on ALL data will be even larger.

### Fractional atomic coordinates and isotropic or equivalent isotropic displacement parameters (Å^2^)

**Table:**

	*x*	*y*	*z*	*U*_iso_*/*U*_eq_	
Co1	0.0000	0.38310 (6)	0.2500	0.0350 (3)	
O1	0.0464 (3)	0.4910 (2)	0.20630 (10)	0.0408 (8)	
O2	0.0446 (4)	0.3536 (2)	0.16381 (11)	0.0516 (9)	
N1	0.1650 (4)	0.2895 (3)	0.25715 (12)	0.0407 (9)	
N2	0.2189 (6)	0.2259 (4)	0.22795 (17)	0.0756 (15)	
H2	0.1940	0.2265	0.2009	0.091*	
C1	0.1346 (5)	0.5130 (4)	0.13559 (15)	0.0406 (11)	
C2	0.2789 (5)	0.5234 (4)	0.13413 (17)	0.0514 (13)	
C3	0.3346 (6)	0.5847 (5)	0.1010 (2)	0.0731 (18)	
H3	0.4323	0.5955	0.0999	0.088*	
C4	0.2509 (7)	0.6304 (5)	0.0697 (2)	0.0778 (19)	
C5	0.1112 (7)	0.6181 (5)	0.07273 (19)	0.0701 (17)	
H5	0.0537	0.6506	0.0519	0.084*	
C6	0.0485 (5)	0.5603 (4)	0.10495 (16)	0.0501 (13)	
C7	0.3722 (6)	0.4637 (5)	0.16474 (19)	0.0630 (16)	
H7A	0.3170	0.4485	0.1911	0.076*	
C8	0.4125 (7)	0.3596 (5)	0.1447 (2)	0.090 (2)	
H8A	0.3282	0.3214	0.1365	0.135*	
H8B	0.4656	0.3182	0.1654	0.135*	
H8C	0.4696	0.3722	0.1193	0.135*	
C9	0.5041 (7)	0.5219 (6)	0.1784 (3)	0.099 (2)	
H9A	0.4785	0.5884	0.1918	0.149*	
H9B	0.5622	0.5354	0.1532	0.149*	
H9C	0.5561	0.4791	0.1989	0.149*	
C10	0.3147 (9)	0.6955 (6)	0.0337 (3)	0.113 (3)	
H10A	0.2387	0.6935	0.0119	0.135*	
C11	0.4247 (10)	0.6439 (8)	0.0113 (3)	0.143 (4)	
H11A	0.4581	0.6889	−0.0119	0.214*	
H11B	0.3901	0.5780	−0.0007	0.214*	
H11C	0.5013	0.6293	0.0311	0.214*	
C12	0.3206 (11)	0.8054 (6)	0.0431 (2)	0.134 (4)	
H12A	0.3590	0.8429	0.0185	0.200*	
H12B	0.3800	0.8170	0.0681	0.200*	
H12C	0.2266	0.8313	0.0491	0.200*	
C13	−0.1072 (6)	0.5466 (4)	0.10610 (17)	0.0563 (14)	
H13A	−0.1330	0.5261	0.1359	0.068*	
C14	−0.1865 (6)	0.6474 (5)	0.0955 (2)	0.079 (2)	
H14A	−0.1570	0.7032	0.1150	0.118*	
H14B	−0.2866	0.6353	0.0988	0.118*	
H14C	−0.1666	0.6680	0.0659	0.118*	
C15	−0.1521 (8)	0.4578 (6)	0.0765 (2)	0.106 (3)	
H15A	−0.1009	0.3941	0.0839	0.160*	
H15B	−0.1320	0.4769	0.0468	0.160*	
H15C	−0.2521	0.4455	0.0798	0.160*	
C16	0.2320 (6)	0.2617 (4)	0.29427 (18)	0.0594 (15)	
H16	0.2176	0.2921	0.3216	0.071*	
C17	0.3225 (6)	0.1830 (4)	0.2852 (2)	0.0651 (17)	
H17	0.3820	0.1489	0.3050	0.078*	
C18	0.3129 (4)	0.1623 (3)	0.24355 (14)	0.0316 (10)	
H18	0.3642	0.1113	0.2281	0.038*	
C19	0.0713 (5)	0.4460 (4)	0.16973 (15)	0.0375 (11)	

### Atomic displacement parameters (Å^2^)

**Table:**

	*U*^11^	*U*^22^	*U*^33^	*U*^12^	*U*^13^	*U*^23^
Co1	0.0309 (4)	0.0278 (4)	0.0463 (5)	0.000	0.0002 (4)	0.000
O1	0.0410 (18)	0.0424 (18)	0.0391 (18)	0.0000 (14)	0.0033 (15)	0.0010 (15)
O2	0.060 (2)	0.0385 (19)	0.057 (2)	−0.0102 (16)	0.0027 (17)	−0.0018 (16)
N1	0.040 (2)	0.0349 (19)	0.048 (3)	0.0090 (17)	−0.0004 (19)	−0.0039 (19)
N2	0.079 (4)	0.069 (3)	0.079 (4)	−0.004 (3)	0.001 (3)	−0.002 (3)
C1	0.044 (3)	0.039 (3)	0.038 (3)	0.000 (2)	0.008 (2)	0.002 (2)
C2	0.044 (3)	0.052 (3)	0.059 (3)	0.003 (2)	0.014 (3)	0.004 (3)
C3	0.055 (4)	0.070 (4)	0.095 (5)	−0.005 (3)	0.033 (4)	0.012 (4)
C4	0.079 (4)	0.065 (4)	0.090 (5)	0.014 (3)	0.038 (4)	0.032 (4)
C5	0.071 (4)	0.077 (4)	0.063 (4)	0.021 (3)	0.020 (3)	0.030 (3)
C6	0.058 (3)	0.048 (3)	0.045 (3)	0.003 (3)	0.006 (3)	0.010 (3)
C7	0.042 (3)	0.079 (4)	0.068 (4)	−0.001 (3)	0.002 (3)	0.006 (3)
C8	0.081 (5)	0.082 (5)	0.107 (6)	0.023 (4)	−0.008 (4)	0.011 (4)
C9	0.049 (3)	0.116 (6)	0.133 (7)	−0.003 (4)	−0.013 (4)	−0.012 (5)
C10	0.113 (6)	0.087 (6)	0.138 (7)	0.013 (5)	0.071 (6)	0.051 (5)
C11	0.138 (8)	0.177 (10)	0.113 (7)	0.020 (7)	0.069 (6)	0.060 (7)
C12	0.221 (11)	0.100 (6)	0.079 (5)	−0.080 (7)	0.038 (6)	0.004 (5)
C13	0.052 (3)	0.069 (4)	0.048 (3)	0.002 (3)	0.000 (3)	0.008 (3)
C14	0.054 (4)	0.099 (5)	0.084 (5)	0.023 (4)	0.002 (3)	0.025 (4)
C15	0.076 (5)	0.121 (7)	0.122 (7)	−0.009 (5)	−0.014 (5)	−0.031 (5)
C16	0.054 (3)	0.069 (4)	0.055 (3)	0.024 (3)	−0.008 (3)	−0.007 (3)
C17	0.046 (3)	0.062 (4)	0.088 (5)	0.026 (3)	−0.006 (3)	0.017 (3)
C18	0.034 (2)	0.0270 (19)	0.034 (3)	0.0153 (18)	0.002 (2)	0.0022 (19)
C19	0.032 (2)	0.045 (3)	0.036 (3)	0.000 (2)	−0.004 (2)	0.001 (2)

### Geometric parameters (Å, °)

**Table:**

Co1—O1^i^	1.993 (3)	C8—H8C	0.9800
Co1—O1	1.993 (3)	C9—H9A	0.9800
Co1—N1	2.000 (4)	C9—H9B	0.9800
Co1—N1^i^	2.000 (4)	C9—H9C	0.9800
O1—C19	1.303 (5)	C10—C11	1.431 (10)
O2—C19	1.223 (5)	C10—C12	1.437 (10)
N1—N2	1.328 (6)	C10—H10A	1.0000
N1—C16	1.375 (6)	C11—H11A	0.9800
N2—C18	1.310 (6)	C11—H11B	0.9800
N2—H2	0.8800	C11—H11C	0.9800
C1—C2	1.394 (7)	C12—H12A	0.9800
C1—C6	1.403 (7)	C12—H12B	0.9800
C1—C19	1.499 (6)	C12—H12C	0.9800
C2—C3	1.404 (7)	C13—C15	1.527 (8)
C2—C7	1.518 (7)	C13—C14	1.534 (8)
C3—C4	1.397 (9)	C13—H13A	1.0000
C3—H3	0.9500	C14—H14A	0.9800
C4—C5	1.355 (9)	C14—H14B	0.9800
C4—C10	1.529 (8)	C14—H14C	0.9800
C5—C6	1.388 (7)	C15—H15A	0.9800
C5—H5	0.9500	C15—H15B	0.9800
C6—C13	1.507 (8)	C15—H15C	0.9800
C7—C8	1.522 (8)	C16—C17	1.360 (7)
C7—C9	1.531 (8)	C16—H16	0.9500
C7—H7A	1.0000	C17—C18	1.331 (7)
C8—H8A	0.9800	C17—H17	0.9500
C8—H8B	0.9800	C18—H18	0.9500
			
O1^i^—Co1—O1	92.32 (18)	C11—C10—C12	121.6 (8)
O1^i^—Co1—N1	121.02 (14)	C11—C10—C4	113.9 (6)
O1—Co1—N1	108.28 (14)	C12—C10—C4	113.5 (7)
O1^i^—Co1—N1^i^	108.28 (14)	C11—C10—H10A	101.2
O1—Co1—N1^i^	121.02 (14)	C12—C10—H10A	101.2
N1—Co1—N1^i^	106.5 (2)	C4—C10—H10A	101.2
C19—O1—Co1	109.7 (3)	C10—C11—H11A	109.5
N2—N1—C16	103.8 (4)	C10—C11—H11B	109.5
N2—N1—Co1	126.8 (3)	H11A—C11—H11B	109.5
C16—N1—Co1	128.4 (3)	C10—C11—H11C	109.5
C18—N2—N1	113.2 (5)	H11A—C11—H11C	109.5
C18—N2—H2	123.4	H11B—C11—H11C	109.5
N1—N2—H2	123.4	C10—C12—H12A	109.5
C2—C1—C6	121.6 (5)	C10—C12—H12B	109.5
C2—C1—C19	118.8 (4)	H12A—C12—H12B	109.5
C6—C1—C19	119.5 (4)	C10—C12—H12C	109.5
C1—C2—C3	117.2 (5)	H12A—C12—H12C	109.5
C1—C2—C7	121.3 (5)	H12B—C12—H12C	109.5
C3—C2—C7	121.4 (5)	C6—C13—C15	110.6 (5)
C4—C3—C2	122.1 (6)	C6—C13—C14	113.0 (5)
C4—C3—H3	118.9	C15—C13—C14	110.7 (5)
C2—C3—H3	118.9	C6—C13—H13A	107.4
C5—C4—C3	118.2 (5)	C15—C13—H13A	107.4
C5—C4—C10	120.9 (7)	C14—C13—H13A	107.4
C3—C4—C10	120.9 (7)	C13—C14—H14A	109.5
C4—C5—C6	122.9 (6)	C13—C14—H14B	109.5
C4—C5—H5	118.5	H14A—C14—H14B	109.5
C6—C5—H5	118.5	C13—C14—H14C	109.5
C5—C6—C1	117.9 (5)	H14A—C14—H14C	109.5
C5—C6—C13	120.7 (5)	H14B—C14—H14C	109.5
C1—C6—C13	121.3 (5)	C13—C15—H15A	109.5
C2—C7—C8	109.3 (5)	C13—C15—H15B	109.5
C2—C7—C9	114.9 (5)	H15A—C15—H15B	109.5
C8—C7—C9	109.2 (5)	C13—C15—H15C	109.5
C2—C7—H7A	107.7	H15A—C15—H15C	109.5
C8—C7—H7A	107.7	H15B—C15—H15C	109.5
C9—C7—H7A	107.7	C17—C16—N1	108.3 (5)
C7—C8—H8A	109.5	C17—C16—H16	125.8
C7—C8—H8B	109.5	N1—C16—H16	125.8
H8A—C8—H8B	109.5	C18—C17—C16	107.9 (5)
C7—C8—H8C	109.5	C18—C17—H17	126.0
H8A—C8—H8C	109.5	C16—C17—H17	126.0
H8B—C8—H8C	109.5	N2—C18—C17	106.8 (4)
C7—C9—H9A	109.5	N2—C18—H18	126.6
C7—C9—H9B	109.5	C17—C18—H18	126.6
H9A—C9—H9B	109.5	O2—C19—O1	121.5 (4)
C7—C9—H9C	109.5	O2—C19—C1	122.0 (4)
H9A—C9—H9C	109.5	O1—C19—C1	116.5 (4)
H9B—C9—H9C	109.5		
			
O1^i^—Co1—O1—C19	−174.0 (3)	C2—C1—C6—C13	178.5 (5)
N1—Co1—O1—C19	62.1 (3)	C19—C1—C6—C13	1.2 (8)
N1^i^—Co1—O1—C19	−61.0 (3)	C1—C2—C7—C8	90.8 (7)
O1^i^—Co1—N1—N2	−169.3 (4)	C3—C2—C7—C8	−84.0 (7)
O1—Co1—N1—N2	−64.9 (4)	C1—C2—C7—C9	−146.0 (6)
N1^i^—Co1—N1—N2	66.7 (4)	C3—C2—C7—C9	39.2 (8)
O1^i^—Co1—N1—C16	23.7 (5)	C5—C4—C10—C11	−130.1 (9)
O1—Co1—N1—C16	128.1 (4)	C3—C4—C10—C11	51.9 (11)
N1^i^—Co1—N1—C16	−100.4 (5)	C5—C4—C10—C12	85.1 (11)
C16—N1—N2—C18	−0.3 (6)	C3—C4—C10—C12	−92.8 (10)
Co1—N1—N2—C18	−169.9 (3)	C5—C6—C13—C15	82.9 (7)
C6—C1—C2—C3	1.0 (8)	C1—C6—C13—C15	−95.5 (6)
C19—C1—C2—C3	178.4 (5)	C5—C6—C13—C14	−41.9 (8)
C6—C1—C2—C7	−174.0 (5)	C1—C6—C13—C14	139.7 (5)
C19—C1—C2—C7	3.4 (8)	N2—N1—C16—C17	0.1 (6)
C1—C2—C3—C4	−2.5 (9)	Co1—N1—C16—C17	169.4 (4)
C7—C2—C3—C4	172.5 (6)	N1—C16—C17—C18	0.2 (7)
C2—C3—C4—C5	2.8 (10)	N1—N2—C18—C17	0.4 (6)
C2—C3—C4—C10	−179.2 (6)	C16—C17—C18—N2	−0.4 (6)
C3—C4—C5—C6	−1.6 (11)	Co1—O1—C19—O2	11.3 (5)
C10—C4—C5—C6	−179.6 (6)	Co1—O1—C19—C1	−168.4 (3)
C4—C5—C6—C1	0.2 (9)	C2—C1—C19—O2	−93.1 (6)
C4—C5—C6—C13	−178.2 (6)	C6—C1—C19—O2	84.3 (6)
C2—C1—C6—C5	0.1 (8)	C2—C1—C19—O1	86.6 (6)
C19—C1—C6—C5	−177.3 (5)	C6—C1—C19—O1	−95.9 (5)

Symmetry codes: (i) −*x*, *y*, −*z*+1/2.
